# Adipose-Derived Mesenchymal Stem Cell (MSC) Immortalization by Modulation of *hTERT* and *TP53* Expression Levels

**DOI:** 10.3390/jpm13111621

**Published:** 2023-11-20

**Authors:** Aigul R. Rakhmatullina, Rimma N. Mingaleeva, Dina U. Gafurbaeva, Olesya N. Glazunova, Aisylu R. Sagdeeva, Emil R. Bulatov, Albert A. Rizvanov, Regina R. Miftakhova

**Affiliations:** 1 Institute of Fundamental Medicine and Biology, Kazan Federal University, 420008 Kazan, Russia; 2 Shemyakin-Ovchinnikov Institute of Bioorganic Chemistry, Russian Academy of Sciences, 117997 Moscow, Russia

**Keywords:** mesenchymal stem cell, tumor stroma, immortalization, MSC, MSC differentiation

## Abstract

Mesenchymal stem cells (MSCs) are pivotal players in tissue repair and hold great promise as cell therapeutic agents for regenerative medicine. Additionally, they play a significant role in the development of various human diseases. Studies on MSC biology have encountered a limiting property of these cells, which includes a low number of passages and a decrease in differentiation potential during in vitro culture. Although common methods of immortalization through gene manipulations of cells are well established, the resulting MSCs vary in differentiation potential compared to primary cells and eventually undergo senescence. This study aimed to immortalize primary adipose-derived MSCs by overexpressing human telomerase reverse transcriptase (*hTERT*) gene combined with a knockdown of *TP53*. The research demonstrated that immortalized MSCs maintained a stable level of differentiation into osteogenic and chondrogenic lineages during 30 passages, while also exhibiting an increase in cell proliferation rate and differentiation potential towards the adipogenic lineage. Long-term culture of immortalized cells did not alter cell morphology and self-renewal potential. Consequently, a genetically stable line of immortalized adipose-derived MSCs (iMSCs) was established.

## 1. Introduction

Mesenchymal stem cells (MSCs) have become important cellular models for fundamental studies of various human diseases. MSCs are widely used as preclinical models for immunological and degenerative disorders, involved in cancer and tissue repair studies [1]. Human MSCs can be isolated from various tissues, including bone marrow, adipose tissue, skin, muscles, and others [2]. The International Society for Cellular Therapy (ISCT) has highlighted several identification criteria for cells to be recognized as MSCs [3]:

– MSCs are plastic-adherent when cultured under standard conditions;
– MSCs are able to differentiate into osteoblasts, adipocytes, and chondroblasts when cultured under specific conditions;
– MSCs express CD90, CD105, and CD73, and lack hematopoietic antigen expression (CD45, CD34, CD14, CD11b, CD79α, or CD19) and histocompatibility antigen (HLA-DR).

In vitro, human MSCs easily adapt to cultivation conditions and initially demonstrate good proliferation rates [4]. However, regardless of tissue origin and/or age, and sex of the donor, during in vitro cell expansion, MSCs undergo aging and significantly reduce cell growth after a limited number of cell passages [4,5]. The term “cellular senescence” is associated with the Hayflick limit, which is the maximum number of times a cell can divide before entering a state of irreversible growth arrest known as “replicative senescence”, ultimately resulting in a decrease in proliferation. Immortalization through genetic manipulation is a rational approach to extend proliferation and bypass normal cellular senescence. The most widely used method for cell immortalization is based on the overexpression of human telomerase reverse transcriptase (*hTERT*) gene or the expression of viral genes—*E6/E7* oncogenes of human papillomavirus (HPV) or SV40 large T antigen (SV40 *L-Tag*) [6]. The first method leads to telomerase activation through the introduction of *hTERT*, which extends the telomeres that shorten with each cell division. Immortalization of MSCs by HPV oncogenes is based on the expression of viral oncoproteins E6 and E7, which can inactivate p53 and pRb, respectively. This inactivation results in the release of the cell cycle arrest and allows cells to bypass senescence and enter an indefinite proliferative state. Similarly to HPV, p53 and pRb inactivation is the main step in SV40-mediated immortalization, but viral proteins also interact with multiple transcriptional co-activators, such as p300 and CBP [7]. 

The results of several studies have shown that modulation at the single gene level is insufficient for cell immortalization; eventually, cellular aging may occur [8,9]. For example, a study by Balducci and co-authors discovered that *hTERT* alone was not effective in immortalizing human adipose-derived stromal cells. However, when the introduction of *hTERT* was combined with either HPV16 *E6/E7* or SV40 *L-Tag*, the cells were successfully immortalized. Interestingly, a reduction in differentiation properties was observed for the resulting cell lines, and was more prominent in *hTERT*/SV40 cells. Furthermore, the combination of *hTERT* with viral genes led to genomic instability, including chromosomal abnormalities such as aneuploidy and structural abnormalities, detected in some of the immortalized cells [10]. At the same time, the introduction of *hTERT* alone has been found to result in fewer changes in cell phenotype and karyotype when compared to the introduction of SV40 *L-Tag* alone [11,12]. 

In general, an effective immortalization strategy should involve both the elimination of p53 or pRB-mediated terminal proliferation and the stimulation of the *hTERT* maintenance mechanism. However, the use of viral proteins is accompanied by their multi-target nature and may lead to genomic instability or alterations in the characteristics of the obtained immortalized cells. Disruption of *TP53* expression with small interfering RNA (siRNA) together with *hTERT* overexpression was successfully combined for the immortalization of primary human ovarian epithelial cells [13]. The resulting cell lines were not tumorigenic in Nude mice and did not form colonies in soft agar assay. The cell karyotype was accessed at passage 50 and indicated that 71.4% to 95.2% of cells were diploid, while only 27.5% of *hTERT*/SV40 cells retained diploidy [13].

A similar strategy with stable siRNA-mediated *TP53* knockdown was applied to immortalize bone marrow-derived MSCs (bmMSCs) [14]. Again, silencing of *TP53* alone was not sufficient for bmMSCs immortalization. *TP53* knocked down–*hTERT* overexpressing bmMSCs preserved the morphology of primary cells for more than 13 months of continuous cultivation. Immortalized cells showed a surface antigen profile similar to that of primary MSCs, a gene expression profile, and retained differentiation potential.

The involvement of p53 in cell senescence induction, differentiation regulation, growth factor production, and cell motility highlights the importance of considering its potential role in the stroma within the tumor microenvironment. There have been reports of *TP53* mutations in the stroma cells of breast cancer samples, and it has been observed that both *TP53* mutation and p53 expression in stromal fibroblasts are linked to lymph node metastasis [15]. Mutations in *TP53* in stroma cells have evidently contributed to epithelial–stromal cross-talk in the carcinogenesis of some primary breast tumors [16]. The status of stromal p53 affected the recruitment of MSCs to solid tumors by regulating CXCL12 production and other mechanisms [17]. Stromal fibroblasts with p53 loss enhanced tumor growth and were associated with a decreased latency of tumor formation in mice [18]. P53-deficient stromal cells in the tumor microenvironment exerted a tumor-promoting effect, and also reduced the ability to improve cancer cell survival during chemotherapy treatment [19,20]. The consequences of p53 loss are attributed to the extensive and fundamental range of biological functions in which p53 is typically involved.

In this study, we aimed to generate a genetically stable immortalized cell line of MSCs for long-term studies in the area of the tumor microenvironment. We used the combination of *hTERT* overexpression with short hairpin RNA (shRNA)-mediated knockdown of *TP53* for the first time to immortalize adipose-derived MSC (adMSC). Cell proliferation, differentiation potential, and self-renewal were assessed following the immortalization of adMSCs.

## 2. Materials and Methods

### 2.1. MSC Isolation

AdMSCs were obtained previously during liposuction [21]. MSCs were isolated from a healthy Caucasian female, testing negative for hepatitis B and C viruses and human immunodeficiency virus. Written informed consent was obtained from the donor following the approval of the local ethic committees of the Republic Clinical Hospital, Russia.

### 2.2. Cell Culture

Primary cells were cultured in a humidified atmosphere with 5% CO_2_ at 37 °C in α-MEM cell culture medium (Paneco Ltd., Moscow, Russia) supplemented with 10% fetal bovine serum (FBS, Biosera, Cholet, France), 2 mM L-glutamine (Paneco Ltd., Russia), and 1% penicillin/streptomycin (Paneco Ltd., Moscow, Russia).

### 2.3. Immunophenotypic Characterization of iMSC

MSC surface antigens were examined using the BD Stemflow™ Human MSC Analysis Kit (BD Bioscience, Franklin Lakes, NJ, USA) according to the manufacturer’s protocol. Stained cells were analyzed on a BD FACS Aria III (BD Bioscience, USA) flow cytometer.

### 2.4. Viral Vectors

For retrovirus assembly, the pBabe-hygro-*hTERT* plasmid encoding the *hTERT* gene and the hygromycin B resistance gene (Cat. No. 1773, AddGene, Watertown, MA, USA), packaging plasmids pUMVC (Cat. No. 8449, AddGene, USA), and pCMV-VSV-G (Cat. No. 8454, AddGene, USA) were used.

Lentivirus assembly was produced by the pLVUHshp53-GFP vector plasmid (Cat. No. 11653, AddGene, USA), encoding short hairpin RNA against the *TP53* and containing the insert gene for green fluorescent protein (GFP), and packaging plasmids psPAX2 (Cat. No. 12260, AddGene, USA) and pCMV-VSV-G. 

Retroviral and lentiviral particles were produced using calcium phosphate transfection of 293FT cells.

The transfection was performed for 16 h. The conditioned media containing viral particles were harvested twice—24 and 48 h after transfection. The collected conditioned media was centrifuged at 3000 rpm for 15 min, filtered through a 0.45 μm Syringe 30 mm PES filters (Jet Biofil, Guangzhou, China), and the viral particles were concentrated in an Optima™ L-90K ultracentrifuge using Ultra-Clear ™ tubes (tubes 1 × 31/2 in and 25 × 89 mm) and rotor SW28 (Beckman Coulter Inc., Brea, CA, USA) for 2 h at 26,000 rpm and 4 °C. Then, the viral particles were resuspended in the DMEM nutrient medium and stored at −80 °C.

### 2.5. Transduction of adMSCs

Immortalized MSCs were obtained as a result of two consecutive transductions: retroviral and lentiviral. Cells at the second passage were seeded at a density of 1 × 10^4^ per well into a dish with a surface area of 3.5 cm^2^ and grown to 60% confluence under standard conditions. Retrovirus hygro-*hTERT* and 8 µg/mL of protamine sulfate for a final volume of 500 µL of Dulbecco’s modified Eagle’s medium (DMEM, Paneco Ltd., Russia) were added to the cells. Transduction was performed for 18 h. Then, the medium was replaced with the culture medium and 48 h later, transduction with lentivirus shp53-GFP was performed similarly to retrovirus transduction. Control MSCs were transduced using a lentiviral vector encoding pWPT-GFP (Cat. No. 12255, AddGene, USA).

The selection of effectively transduced cells (by hygro-*hTERT*) was carried out using 50 μg/mL of hygromycin for two weeks. During the next step, cells with high (>100 fold) levels of GFP expression were sorted using a FACS Aria III flow cytometer (BD Bioscience, USA). The isolated immortalized MSC (iMSC) cell population was further characterized immunophenotypically, as described above.

### 2.6. Cell Proliferation Assay

The effects of transduction on cell proliferation were assessed using the MTT assay (Promega, Madison, WI, USA), according to the manufacturer’s protocol. Initially, cells were seeded at a density of 2 × 10^3^ cells per well onto 96 well plates in a full growth medium. The optical density values were measured at 560 nm with a reference to 690 nm using Tecan Infinity 2000 (Tecan Group Ltd., Männedorf, Swizerland). The number of cells was recalculated using the calibration curve data. To construct the calibration curve, additional cells were seeded in a range of 0–8000 cells per well. All sample were presented in three technical replicates with three biological experiments conducted.

### 2.7. Induction of Adipogenic Differentiation of iMSCs 

Osteo-, chondro- and adipogenic differentiation of iMSCs were induced as described previously [22].

The induction of adipogenic differentiation was performed according to the manufacturer’s protocols using the StemPro**^®^** Adipogenesis Differentiation Kit (Cat. No. A10070-01, Invitrogen, Waltham, MA, USA). Briefly, *hTERT*-shp53-transduced MSCs were cultured in a 6-well plate under standard conditions. When 80% of the cell growth density was reached, the culture medium was replaced with the differentiation medium, which was changed every three days. After 14 days, cells were fixed with a 4% formalin solution (BioVitrum, St. Petersburg, Russia) for 5 min, washed with deionized water, and then stained with Oil Red O (Cat. No. O0625, Sigma-Aldrich, St. Louis, MI, USA) for 15 min.

### 2.8. Induction of Osteogenic Differentiation of iMSCs 

The induction of osteogenic differentiation was performed according to the manufacturer’s protocols using StemPro**^®^** Osteogenesis Differentiation Kit (Cat. No. A10072-01, Invitrogen, USA). Cells were cultured in a 6-well plate under standard conditions. When 80% of the cell growth density was reached, the culture medium was changed to a differentiation medium, which was changed every four to five days. After 21 days, cells were fixed with a 4% solution of paraformaldehyde (BioVitrum, Russia) for 30 min, washed with deionized water, and then stained with freshly prepared Alizarin red S solution (company) for 5–10 min.

### 2.9. Induction of Chondrogenic Differentiation of iMSCs

The induction of chondrogenic differentiation was performed according to the manufacturer’s protocols using the StemPro**^®^** Chondrogenesis Differentiation Kit (Cat. No. A10071-01, Invitrogen, USA).

Cells (2 × 10^3^) in a 5-µL drop were seeded in the center of the well in a 96-well plate. After 4 h of incubation, a differentiation medium was added to the cells. The differentiation medium was changed every four days for 28 days. Differentiated cells were fixed with a 4% formalin (BioVitrum, Russia) solution for 30 min and stained with Alcian Blue solution (Cat. No. A5268, Sigma-Aldrich, USA) for 20–30 min. 

Cell differentiation efficacy was analyzed under the Nikon Eclipse TS100-F (Nikon Instruments Inc., Tokyo, Japan) microscope using ×10, ×40 magnifications. The differentiation capability of iMSC was assessed by counting the proportion of stained versus unstained cells.

### 2.10. MSC Sphere Formation Assay

The sphere formation potential was analyzed by seeding iMSC cells at a density of 1 × 10^4^ into non-treated culture dishes in DMEM/F-12 (Paneco Ltd., Russia) medium supplemented with 2× B27 (Paneco Ltd., Russia), 40 ng/mL EGF (Sci-store Ltd., Moscow, Russia), and 40 ng/mL FGF2 (Sci-store Ltd., Russia). Cells were grown for 7 days, and the number of spheres (>40 um) formed was counted. The sphere formation efficacy (%) was calculated as a ratio of the number of spheres formed to the number of cells seeded. Spheres were visualized, and pictures were captured with the Nikon Eclipse TS2R (Japan) microscope. After 7 days in culture, spheres were collected and centrifuged at 400× *g*. 

### 2.11. Analysis of Cytokine Levels in Conditioned Media

The level of cytokines was evaluated in sphere-conditioned media using a multiplex technology using the MILLIPLEX MAP Human Cytokine/Chemokine Magnetic Bead Panel, 41 plex kit (#HCYTMAG-60K-PX41, Merck Millipore, Burlington, MA, USA) in accordance with the recommendations of the manufacturer. For each sample, 25 microliters were used to determine the analyte concentrations. Cytokines levels were analyzed by collecting a minimum of 50 beads per analyte. Median fluorescence intensities were collected using the MAGPIX System with xPONENT 4.2 (Luminex, Austin, TX, USA). Data were analyzed using Bio-Plex Manager 6.1 Software.

### 2.12. Statistical Processing

To compare the cell count between the control cells and iMSCs, a Student’s *t*-test was used for statistical data analysis, as the normality test (Shapiro-Wilk) was passed. The difference was considered significant at *p* < 0.05.

## 3. Results

### 3.1. Immortalized MSCs Were Obtained by Modulation of hTERT and p53 Levels

**Figure 1:**
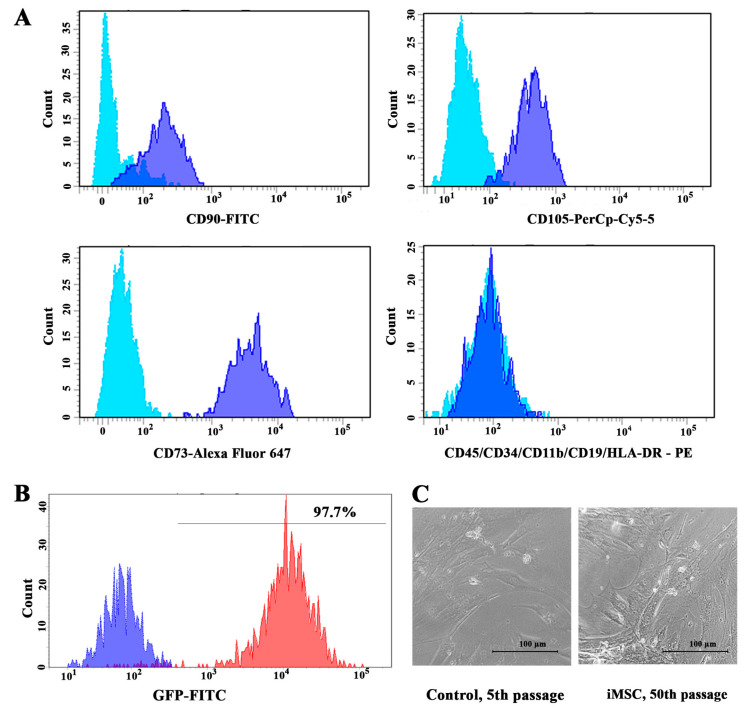
Phenotypic characterization of adMSCs: (**A**) Immunophenotypic analysis of adipose-derived cells from flow cytometry-generated histograms. Light blue curves represent isotypic control and blue curves represent fluorescence peaks. (**B**) Flow cytometry histogram of GFP-FITC fluorescence signal. The blue curve represents negative control—non-immortalized cells; red curve represents iMSCs. (**C**) Cell morphology of iMSC cells at 50th passage did not change compared to control cells at 5th passage.

The immortalization by *hTERT* overexpression and *TP53* inactivation is primarily aimed at halting replicative senescence. In our study, the transduction of two vectors carrying hygro-*hTERT* and shp53-GFP inserts, respectively, was performed at low (2nd) cell passages owing to the heightened capacity for growth and proliferation. Initially, the expression of surface markers of isolated MSCs was analyzed using flow cytometry to confirm cell identity. All cells lacked the expression of hematopoietic markers and were positive for the expression of differentiation clusters CD90, CD105, and CD73 (Figure 1A). 

The pLVUHshp53-GFP vector plasmid was used to co-transduce shRNA against *TP53* with the green fluorescent protein (GFP) gene. Control MSCs were transformed by lentiviral particles expressing GFP alone. The efficacy of *TP53* inactivation was assessed by GFP expression using flow cytometry (Figure 1B), and fluorescent microscopy (Supplementary Figure S1A). Furthermore, transduced cells were sorted based on GFP intensity. Following *hTERT* transduction, cells were subjected to hygromycin selection, as the pBabe-hygro-*hTERT* plasmid encoded hygromycin B resistance gene. No significant changes in cell morphology were detected between immortalized and control cells. Moreover, the morphology of the iMSCs did not change during long-term in vitro cultivation (Figure 1C). 

### 3.2. Immortalization Increased Cell Proliferation Rate and Sustained Differentiation of MSCs

**Figure 2:**
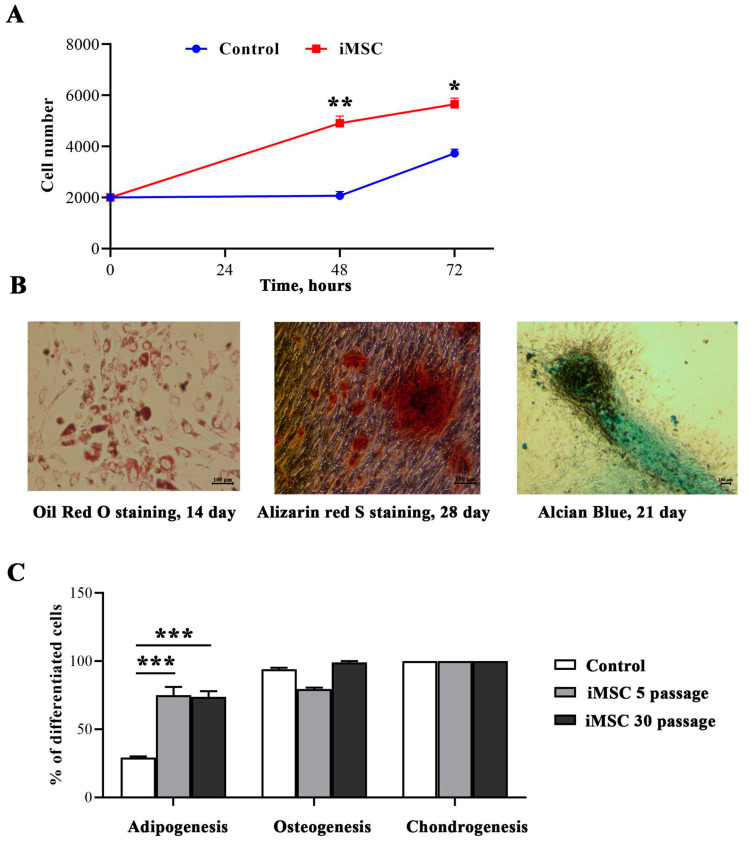
Functional characterization of iMSCs in comparison to control cells: (**A**) Diagram representing the MTT-assay for cell proliferation rate of iMSCs and control cells. Three replicates were obtained for each cell line. Cells were collected at the 5th passage for control cells and at the 5th and 50th passages for iMSCs. Error bars show mean ± SD; *-*p* < 0.05, **-*p* < 0.01. (**B**) Representative pictures of adipogenesis with Oil red O dye staining neutral lipids in cells; osteogenesis with Alizarin red S dye staining calcium deposits in cells; chondrogenesis with Alcian dye staining sulfated proteoglycan in cells. (**C**) The histogram of differentiated cell count of iMSCs at the 5th and 30th passages compared to control. The data is presented as a mean value ± SD; ***-*p* < 0.001.

The proliferation assay showed that by 72 h of culture, iMSC number increased threefold, and the proliferation rate was significantly higher compared to control non-immortalized cells (*p* = 0.0147) (Figure 2A).

To investigate whether immortalization affected cell differentiation potential, adipogenic, osteogenic, and chondrogenic differentiation of iMSCs and non-immortalized MSCs was assessed. We also examined whether long-term culture changed differentiation potential in the immortalized cells at passages 5 and 30.

In our study, the ability to differentiate into the adipogenic lineage was significantly increased in the immortalized cells compared to primary MSCs (Figure 2B,C). To evidence lipid inclusions in cells, oil red O staining was performed on the 14th day of differentiation. The differentiation efficiency of the primary MSCs was 29.2 ± 0.8%, while lipid inclusions were observed in 74.9 ± 6.1% of iMSCs (*p* < 0.001). The iMSCs at the 30th passage retained their differentiation potential: the efficiency was 73.8 ± 4.1% (*p* < 0.001). 

Cell culture in an osteogenic media contributed to the formation of calcium deposits in the intercellular space. To visualize the differentiation of stem cells into osteoblasts, bone nodules were stained with Alizarin red dye (Figure 2B). Differentiation in the osteogenic direction was 94.2 ± 3% for control MSCs, 79.5 ± 4.5% for iMSCs at the 5th passage, and 99.1 ± 3.8% for iMSCs at the 30th passage (Figure 2C). 

Induction of cell differentiation into chondrocytes was visually determined by staining proteoglycan sulfates of the extracellular matrix with Alcian Blue at the 5th and 30th passages of iMSC (Figure 2B). The deposits of proteoglycan sulfates acquired a blue color, which, in turn, confirmed the success of chondrogenesis in cells. The ability to differentiate into chondroblasts was shown for all studied cell lines by qualitative analyses. We did not observe differences between the levels of chondrogenesis at the early and late passages of iMSC (Figure 2C).

### 3.3. iMSCs Retain Self-Renewal Ability

**Figure 3:**
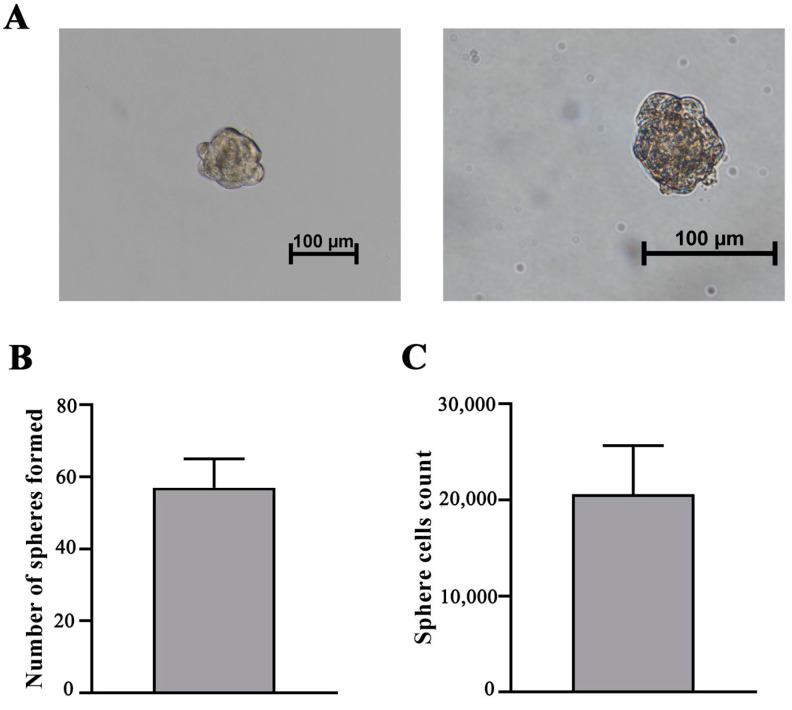
Sphere formation efficacy evaluation. (**A**) Sphere formation of iMSC-c2 after cultivation in serum-free cell culture medium at 200× (**left**) and 400× (**right**) magnification. (**B**) Histogram of sphere count when initially 1 × 10^4^ cells were seeded. Bars show the mean ± SD of three biological replies per microscopic field at 100× magnification. (**C**) Histogram displaying cell number in iMSC spheres. Bars show the mean ± SD of three biological replicates.

To evaluate the ability of iMSCs to form spheres, the cells were cultured in sphere-formation media for a period of 7 days. iMSCs displayed a high efficacy of sphere formation (Figure 3A). The mean number of spheres in culture was 57 ± 8, and the efficacy of sphere formation was 0.5%. On average, 20,600 ± 5660 cells were detected in spheres (Figure 3B,C). These results suggest that the immortalization of adMSCs by *hTERT* and p53 modulation did not have an impact on the self-renewal ability of MSCs. 

Furthermore, the concentrations of key regulatory factors, such as cytokines and growth factors, were assessed in the conditioned media of the spheres (Table 1). The analysis revealed high levels of angiogenic factors, including vascular endothelial growth factor (VEGF), interleukin-8 (IL-8), and monocyte chemoattractant protein 1 (MCP-1) in the conditioned media of iMSC-derived spheres. 

Among the evaluated immunomodulatory factors, relatively high levels of IL-8, IL-6, MCP3, granulocyte colony-stimulating factor (G-CSF), C-X-C motif chemokine ligand 10 (CXCL10, IP-10), C-X-C motif chemokine ligand 1 (CXCL1, GRO), and regulated upon activation, normal T cell expressed and presumably secreted (RANTES) were detected.

## 4. Discussion

AdMSCs are a valuable resource for regenerative medicine due to their abundance in the human body and ease of accessibility [23]. To overcome the limitations associated with senescence and cell aging, primary stem cell immortalization is utilized. It is important to note that viral gene expression can lead to insertional mutagenesis, resulting in unpredictable gene expression and oncogenic transformation. In contrast, p53 knockdown by siRNA has been previously associated with a low risk of mutagenesis [13]. Moreover, viral gene expression can potentially trigger an immune response. Lui et al. proposed a novel approach for safer MSC immortalization by modulating only human genes. They found that the *hTERT* hyperexpression combined with the *TP53* knockdown method resulted in long-term stable growth and preserved differentiation potential. Moreover, the immortalized MSCs showed no signs of tumorigenicity in vivo [14]. In our study, we confirm that a similar strategy can be applied for adMSC immortalization. 

We demonstrated a higher proliferation rate of immortalized cells compared to non-immortalized cells (Figure 2A). The enhanced proliferative activity may be affected by p53 silencing, as previous research has shown that stem cells transduced with a combination of *hTERT* with p53-targeting SV40 *L-Tag* or HPV *E6/E7* benefit from an increased in vitro expansion rate compared to single *hTERT* overexpressing cells [10]. 

Furthermore, iMSCs retained their potential for differentiation for at least 30 passages (Figure 2B,C). In the presence of specific growth factors, MSCs can differentiate into various cell types. However, the proliferation, differentiation, and immunomodulatory characteristics depend on the initial source of cell isolation [24]. For example, amniotic fluid-derived MSCs proliferate faster than bmMSC [25]. BmMSC display a higher osteogenic proliferation capacity compared to adMSC [26]. Furthermore, adMSC is more efficient in blocking T-cell activation compared to bmMSC and umbilical cord matrix-derived MSC when co-cultured with phytohemagglutinin-activated T-cells [27]. Notably, immortalized bmMSCs display higher differentiation potential towards the osteogenic lineage [28]. It can be assumed that the high rates of adipogenic differentiation are due to the fact that the cells were initially isolated from adipose tissue. 

A novel hierarchical model of MSC self-renewal was proposed by Sarugaser and colleagues, suggesting that a self-renewing multipotent MSC generates more limited self-renewing progenitors, which then progressively lose their ability to differentiate until they are completely restricted to becoming fibroblasts [29]. In vitro sphere formation assay is a commonly used functional test for identifying various types of stem cells, including tumor, neuronal, and embryonic stem cells. Several publications have used sphere-like tests, mainly hanging drop culture or cell aggregation techniques, to obtain MSC aggregates [30,31]. We have adapted a protocol for single stem cell-derived mammosphere formation [32] and obtained iMCS-derived spheres as a result of cell self-renewal (Figure 3). iMSCs displayed high efficacy of sphere formation, indicating the maintenance of self-renewal after immortalization of MSCs. 

A growing amount of evidence suggests that MSCs primarily exert their in vivo effects through paracrine signaling, and the range of factors secreted by MSCs is highly adaptable. Previous research has demonstrated that the immortalization of MSCs can impact the spectrum of secreted proteins [33]. Consistent with our findings, Paprocka and colleagues have observed high levels of MCP-1 and GRO in immortalized cells. Additionally, they have also demonstrated that the expression of angiogenic factors such as VEGF, IL-8, and MCP-1 in the conditioned media of immortalized MSCs may be linked to hypoxia, which eventually occur during sphere formation in our study.

## 5. Conclusions

Adipose tissue can be easily isolated by various methods, such as blepharoplasty, levator muscle resection, and laparotomy [4]. In our study, for the first time, we show that *hTERT* hyperexpression combined with shRNA-driven *TP53* knockdown results in the successful immortalization of adMSC. During expansion, immortalized adMSCs maintain their morphology, as well as their potential for adipogenic, chondrogenic, and osteogenic differentiation, while exhibiting an improved proliferative rate and sphere-forming ability. The establishment of an immortalized adMSC line aims to create an in vitro model for various long-term studies. iMSCs could be utilized for studying the mechanisms underlying normal tissue development and disease progression, as well as for investigating potential therapies for various disorders. Additionally, these cells could serve as a consistent and reliable source of MSCs for regenerative medicine applications.

## Figures and Tables

**Table 1:** Concentrations of key immune regulators in the conditional media of iMSC spheres.

Analytes	C, pg/mL	Analytes	C, pg/mL
Eotaxin	5.2 ± 0.5	IL-7	2.2 ± 1.2
FLT-3L	2.8 ± 0.6	IL-8	176.4 ± 83.2
Fractalkine	13.5 ± 6.7	IL-9	0.0
G-CSF	26.0 ± 19.3	IL12p70	1.0 ± 0.1
GM-CSF	0.4 ± 0.3	IFNA2	5.4 ± 1.4
GRO	21.0 ± 18.5	IFNy	0.2 ± 0.0
IL-10	2.0 ± 0.8	IP-10	16.7 ± 10.2
IL-12p40	0.9 ± 0.3	MCP-1	1080.3 ± 539.6
IL-13	1.2 ± 1.2	MCP-3	109.5 ± 52.4
IL-15	0.2 ± 0.0	MDC	5.7 ± 0.8
IL-17A	0.1 ± 0.0	MIP-1a	0.3 ± 0.0
IL-1RA	2.6 ± 1.1	MIP-1b	1.3 ± 0.0
IL-1a	0.3 ± 0.1	PDGF-AA	0.4 ± 0.0
IL-1b	0.6 ± 0.5	PDGF-AB/BB	2.3 ± 0.0
IL-2	2.3 ± 0.0	RANTES	3.9 ± 1.9
IL-3	0.0 ± 0.0	TGF-a	1.6 ± 0.0
IL-4	2.7 ± 2.0	TNFa	1.2 ± 0.0
IL-5	0.2 ± 0.0	TNFb	1.4 ± 0.0
IL-6	273.2 ± 126.0	VEGF	37.0 ± 10.0
sCD40L	0.1 ± 0.0		

## Data Availability

Data are contained within the article.

## Supplementary Materials

The following supporting information can be downloaded at: https://www.mdpi.com/article/10.3390/jpm13111621/s1, Figure S1: Fluorescent microphotographs of iMSCs.
