# Supplementary material for: Adipose-Derived Mesenchymal Stem Cell (MSC) Immortalization by Modulation of hTERT and TP53 Expression Levels
Source: J Pers Med. 2023 Nov 20;13(11):1621. doi: 10.3390/jpm13111621 (PMC10672200; doi:10.3390/jpm13111621)
Supplement: Supplementary file 1 [file jpm-13-01621-s001.zip › jpm-2684743-supplementary.pdf]

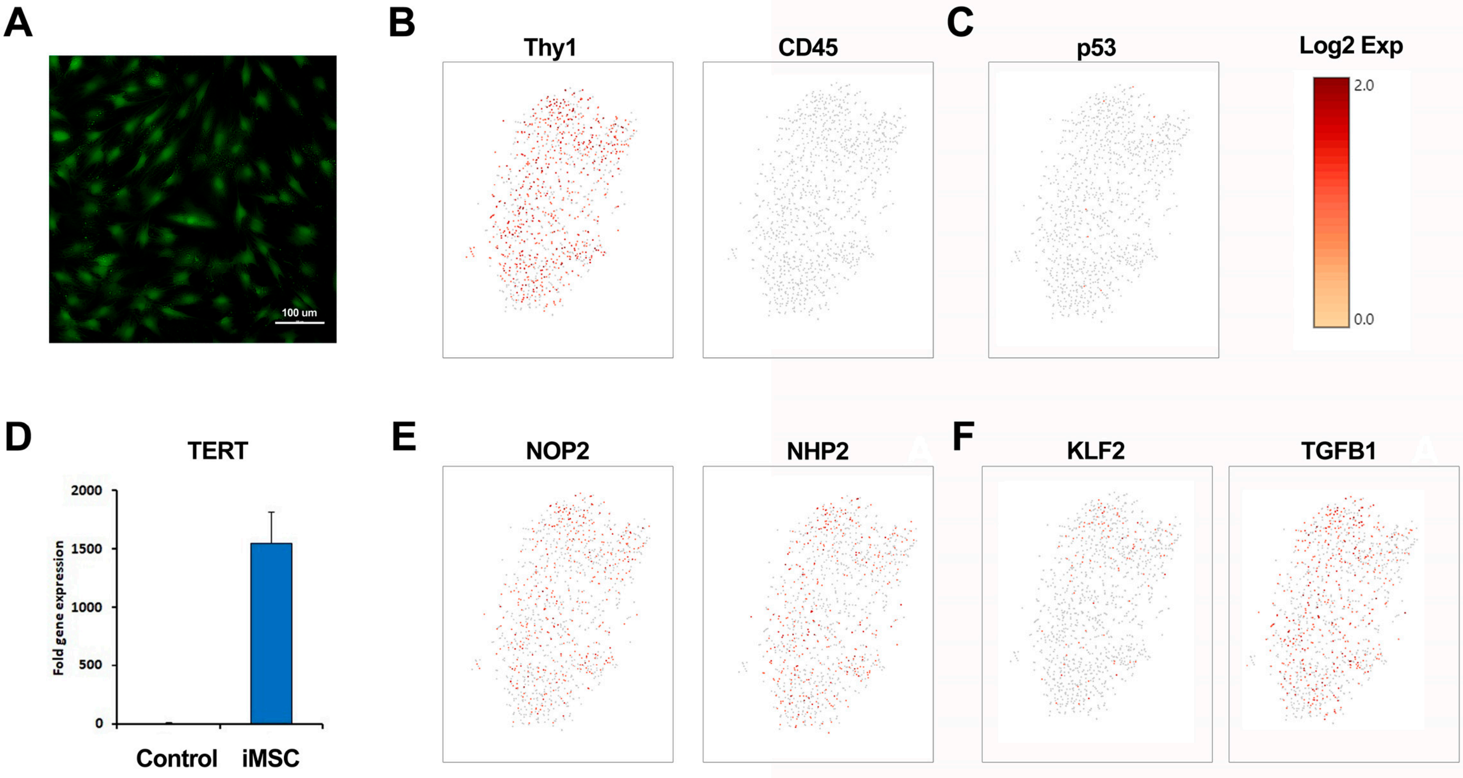

Supplementary figure S1. Fluorescent microphotographs of iMSCs (A). Gene expression profiles from single cells were clustered using tSNE: MSC markers (B), p53 (C), telomerase complex proteins (E) and stem cell markers (F). TERT level in iMSCs (D).
